# Rapid evidence review to inform safe return to campus in the context of coronavirus disease 2019 (COVID-19)

**DOI:** 10.12688/wellcomeopenres.17270.1

**Published:** 2021-10-20

**Authors:** Trisha Greenhalgh, Aris Katzourakis, Tristram D. Wyatt, Stephen Griffin

**Affiliations:** 1 Nuffield Department of Primary Care Health Sciences, University of Oxford, Oxford, OXFORDSHIRE, OX2 6GG, UK; 2 Department of Zoology, University of Oxford, Oxford, OXFORDSHIRE, OX1 3SY, UK; 3 Leeds Institute of Medical Research, University of Leeds, Leeds, YORKSHIRE, LS9 7TF, UK

**Keywords:** SARS-CoV-2, aerosol transmission, hierarchy of controls, higher education policy, infection prevention and control

## Abstract

**Background:** Severe acute respiratory syndrome coronavirus 2 (SARS-CoV-2) is transmitted predominantly through the air in crowded and unventilated indoor spaces among unvaccinated people. Universities and colleges are potential settings for its spread.

**Methods:** An interdisciplinary team from public health, virology, and biology used narrative methods to summarise and synthesise evidence on key control measures, taking account of mode of transmission.

**Results:** Evidence from a wide range of primary studies supports six measures. **Vaccinate** (aim for > 90% coverage and make it easy to get a jab). **Require masks indoors**, especially in crowded settings. If everyone wears well-fitting cloth masks, source control will be high, but for maximum self-protection, respirator masks should be worn.  Masks should not be removed for speaking or singing. **Space people out** by physical distancing (but there is no “safe” distance because transmission risk varies with factors such as ventilation, activity levels and crowding), reducing class size (including offering blended learning), and cohorting (students remain in small groups with no cross-mixing). **Clean indoor air** using engineering controls—ventilation (while monitoring CO _2 _levels), inbuilt filtration systems, or portable air cleaners fitted with high efficiency particulate air [HEPA] filters). **Test asymptomatic staff and students** using lateral flow tests, with tracing and isolating infectious cases when incidence of coronavirus disease 2019 (COVID-19) is high. **Support clinically vulnerable people** to work remotely. There is no direct evidence to support hand sanitising, fomite controls or temperature-taking. There is evidence that freestanding plastic screens, face visors and electronic air-cleaning systems are ineffective.

**Conclusions:** The above six evidence-based measures should be combined into a multi-faceted strategy to maximise both student safety and the continuation of in-person and online education provision. Staff and students seeking to negotiate a safe working and learning environment should collect data (e.g. CO _2 _levels, room occupancy) to inform conversations.

## Introduction

### Severe acute respiratory syndrome coronavirus 2 (SARS-CoV-2) and university life

The United Kingdom (UK) is currently (Autumn 2021) experiencing high and rising levels of coronavirus disease 2019 (COVID-19) cases, and almost all are the highly contagious delta variant ^1^. This variant spread fastest among the 17–24 year age group in June and July 2021 ^2^, likely due to a combination of low vaccination rates in this age group and suboptimal mitigation strategies in schools and colleges. Whilst young people are much less likely to develop severe acute disease from COVID-19 than older people, some will be hospitalised and a few could die ^3^. The incidence of persistent symptoms beyond the acute illness (post-acute or “long” Covid ^4^) is disputed, but a secondary analysis of Office of National Statistics data (published as a preprint) suggests that 4.7% of the 18–24 year age group have some symptoms persisting beyond 12 weeks and 1.1% have symptoms which interfere “a lot” with their daily activities ^5^. University staff and graduate students include older age groups, minority ethnic groups and those with medical conditions, all of which increase the risk of developing serious complications from COVID-19.

For all these reasons, measures to reduce transmission of the virus are needed. Most universities and colleges in the UK now have the infrastructure to implement rapid and frequent testing and support students to isolate when necessary. Since most courses were delivered online in 2020–21, they have also learnt a great deal about how to deliver effective learning online. There is, however, a considerable appetite to return to face-to-face modes of teaching as well as return to traditional levels of socialising, arts and sport.

### The science of SARS-CoV-2 transmission

There is strong and consistent evidence that the main—and perhaps the only significant—mode of transmission of SARS-CoV-2 is through the air ^6, 7^. Indeed, super-spreader events (in which one or a few people infect large numbers of others)—including choir practices, funerals, conferences, gym sessions and other mass indoor events—are likely to be the main drivers of the pandemic ^8^. Higher education includes many preconditions for such super-spreader events, including living and eating communally, lectures and seminars, sports training and competition, arts and singing performances, and socialising.

Some indoor events show no COVID-19 transmission, even when infected people are present, while others are shown in retrospect to have been super-spreader events; this phenomenon is known as heterogeneity or *overdispersion* of transmission dynamics, and is highly relevant to our efforts to control the virus in schools and universities ^9^. Whilst the highest risk of airborne viral transmission occurs with coughing and sneezing, speaking and singing are also high-risk activities ^10, 11^.

**Figure 1.**
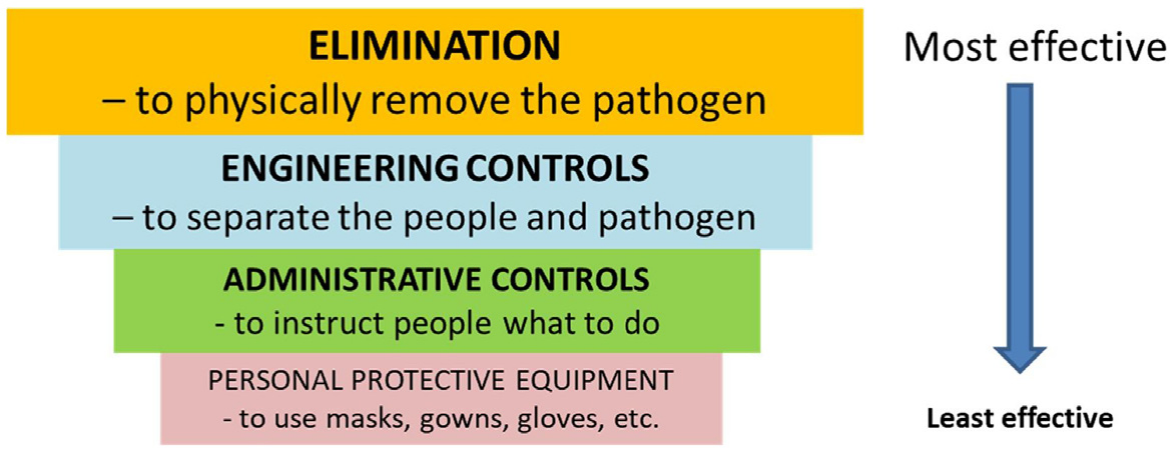
The hierarchy of controls for an infectious pathogen (reproduced under Creative Commons licence from 12).

A landmark paper on minimising risk of airborne transmission (written before effective vaccines had been discovered) used their own adaptation of the US Centers for Disease Control and Prevention’s “hierarchy of controls” ( Figure 1) ^12^. In the sections which follow, we consider all the measures in the hierarchy of controls plus vaccination. We begin with vaccination, masking and administrative controls as these are things which individual university employees may be able to influence. We then discuss engineering controls (ventilation and air filtration).

## Methods

This review, which aimed to produce a synthesis rapidly in time for the new university term, was undertaken in September 2021. We began with sources known to the authors, including a rapid review by Independent SAGE ^1^, and a search of the PubMed database using the terms “SARS-CoV-2”, “COVID-19”, “transmission”, “mitigation”, “school[s]” and “university/ies” (restricted to review articles). Using a method previously shown to be highly efficient for identifying key studies from complex and heterogeneous datasets ^13^, we initially focused on seminal papers (in high-impact journals and highly-cited for their age) and used snowball searching (tracking the article in Google Scholar and pursuing relevant sources from its reference lists) to identify further key studies from these. For specialist subsections that were beyond our own expertise, we undertook further key word searches (e.g. HEPA filters) and consulted with experts in the field. In producing our narrative synthesis of these sources, we prioritised findings that would be useful to inform actual policies in universities.

## Main findings

Direct empirical evidence on mitigation strategies specifically for universities was sparse, but there was much relevant evidence on mitigation measures more generally. Our review suggested that six key measures, which we consider in turn below, are likely to be effective at reducing on-campus spread: vaccination, masking, spacing people out (physical distancing, reducing class size and cohorting), engineering controls (ventilation or filtration of air), a test/trace/isolate policy when COVID-19 incidence is high, and supporting clinically vulnerable people to work remotely. Other widely-promoted measures including sanitising hands, taking temperatures, plastic screens and face visors, were not supported by evidence.

### Encourage vaccination—and make it easy

Vaccines have been a game-changer for COVID-19; they dramatically reduce the incidence of symptomatic disease and risk of transmission of the virus to others; breakthrough infections in vaccinated persons are rare and generally mild ^14^. A recent BMJ review concluded that the most important single intervention for preventing on-campus transmission is vaccination ^15^. These authors suggest that if 90% of staff and students are fully vaccinated, campuses may be able to reopen safely without other measures. However, this 90% cut-off is based on a single preprint modelling study ^16^ that has yet to be peer-reviewed.

The evidence, while sparse, supports strenuous efforts to increase the proportion of staff and students who are fully vaccinated. Most universities are a long way from meeting a 90% target. As of end September 2021, for example, only 58% of 18–24 year olds in England were fully vaccinated ^17^. This is partly because younger age groups were the last to be invited, but also because of relatively high levels of vaccine hesitancy among student age groups, due to a combination of perceived low vulnerability and the “inconvenience” of attending for a jab ^15^. One key measure for improving safety is to make it very easy for people to get a vaccination on campus—for example by locating vaccination hubs close to settings frequented by target groups (e.g. outside lecture theatres or dining halls) and not requiring paperwork that people are unlikely to be carrying.

Vaccination, however, is unlikely to be the sole measure to protect students and staff. Another preprint modelling study, which used the delta variant, found that even at 100% coverage, vaccination was insufficient to eliminate SARS-CoV-2 transmission in a university dormitory setting ^18^. Some vulnerable groups are unable to receive the vaccine or mount an effective immune response to it ^19^. An individual interacting with another group of people in a university setting has no way of knowing what proportion of them are vaccinated. For all these reasons, other measures are likely to be needed for the foreseeable future.

### Everyone should wear masks

Masking has two main effects: reducing emission of the virus by the wearer (“source control”), and protecting the wearer from virus emitted by others ^20– 22^. It also has a third potential effect—reminding us that we are still in a pandemic and signalling to others that we are taking *their* safety seriously ^23^.

Reviews of a wide range of evidence (including laboratory studies and natural experiments) have shown that, broadly speaking, masks are effective—but by no means perfect—for source control ^20– 22^. Masks reduce the amount of virus that gets into the air, and hence the probability that someone else in the room will be infected ^22^. Wearing a mask reduces viral emissions from coughing and sneezing approximately 20-fold ^24^, but around half of all people who transmit the virus have no symptoms at the time (i.e. they are not coughing or sneezing but simply *exhaling* the virus in aerosols) ^25^. Different materials for cloth masks have very different filtration properties ^26^; a well-fitting mask with no leaks round the side is crucial ^27^. A double-layer neck gaiter (bandana) and a medical mask both reduce emission of aerosols by around 60%, but respirator (FFP2 or FFP3, N95) masks are much more effective, blocking up to 99% of aerosols ^28^. Note that face visors reduced aerosol emission by only 5%—i.e. they are ineffective ^28^.

There have been claims that randomised controlled trial (RCT) evidence is the only “robust” way to test the impact of masks. This is incorrect, because most such RCTs are designed only to test the hypothesis that the mask protects the wearer over a short period. Actually, masks work *mainly* by protecting other people, and even a non-statistically significant effect on transmission dynamics (e.g. in lectures) can lead to very large effects over time (for example, if instead of doubling every 9 days, new cases increased by only 1.9-fold, after 180 days cases would be down by 60%).

The above findings support mandating (rather than just encouraging) masking in shared spaces. If everyone is wearing a mask, source control will be high and double-layer cloth masks will be adequate for most healthy people. In one recent Centers for Disease Control and Prevention (CDC) report, US schools without mask mandates in July-August 2021 were 3.5 times more likely to have COVID-19 outbreaks than schools with mandates ^29^. To *protect the wearer* effectively from airborne virus when others in the room are unmasked, a higher grade of filtration is needed, hence in the absence of near-universal use of source control masks, individuals may be left with little choice but to consider respirators ^20^. Those who are clinically vulnerable (hence requiring masks for self-protection) should use respirators in any case.

Perhaps the most persuasive argument for masks in the university context is that if everyone wears one, there is a much lower risk that teaching will need to return to online as a result of rising case numbers.

Since speaking and singing increase emission of aerosols ^10, 11^, masks should be worn continuously indoors and not removed for these activities. The suggestion in some universities that masks should be worn only until people are seated but may be removed thereafter makes no scientific sense. Indeed, because of the airborne nature of the disease, masking is *more* important when in a classroom learning setting (indoors, with others, and with some people talking) than when moving between classrooms (especially if walking alone, outdoors and in silence). Likewise, rules in gyms that masks should be worn when walking between equipment but not when exercising *on* the equipment are nonsensical, since heavy breathing during exercise will increase emission of viral particles ^30, 31^.

The benefit of mask wearing by all is not dependent on the size of a group, so suggestions that masking is needed only above a certain occupancy threshold means that unmasked smaller groups would carry a preventable risk (and also provide a false sense of security).

A major risk setting for transmission of COVID-19 is lunch and tea breaks, since masks must be removed for eating and drinking, and because people often sit at close quarters and talk. To reduce transmission, refreshment breaks should ideally be taken out of doors. If this is not possible, physical distancing should be increased and silence maintained while unmasked. Socialising in breaks could occur, for example, during the walk to the café (while masked) but not while eating.

A few people have a medical reason not to mask (e.g. neurodiverse or anxious) ^32^; in some universities they may obtain a lanyard to indicate they are exempt.

### Space people out (physical distancing, joining remotely, cohorting)

Physical distancing (sometimes called social distancing) is effective at reducing droplet transmission, since droplets fall to the ground within a few feet due to gravity ^33^. Physical distancing also protects against airborne transmission, since most airborne particles are spread via close contact, especially when a person is in the direct stream of someone else’s exhaled breath (think of smelling the garlic on someone’s breath—you might be able to smell it across the room but it is much stronger at close range) ^7^.

Many university guidelines stipulate a specific physical distance such as 1 or 1.5 metres to space desks apart. Whilst this is a useful rule of thumb, a “safe” distance cannot be calculated precisely, since a) airborne particles spread throughout a room within about 30 minutes (and can remain even after the room has been vacated), hence time spent indoors must also be factored in; b) if nobody is wearing a mask, viral emission is considerably greater (hence, close contact is more risky—and conversely if everyone is masked, it is less risky); c) singing or loud talking increases viral transmission (hence, again, close contact is more risky); d) even wide separation will not protect fully against the turbulent jets emitted when a symptomatic person coughs or sneezes ^33^.

**Figure 2.**
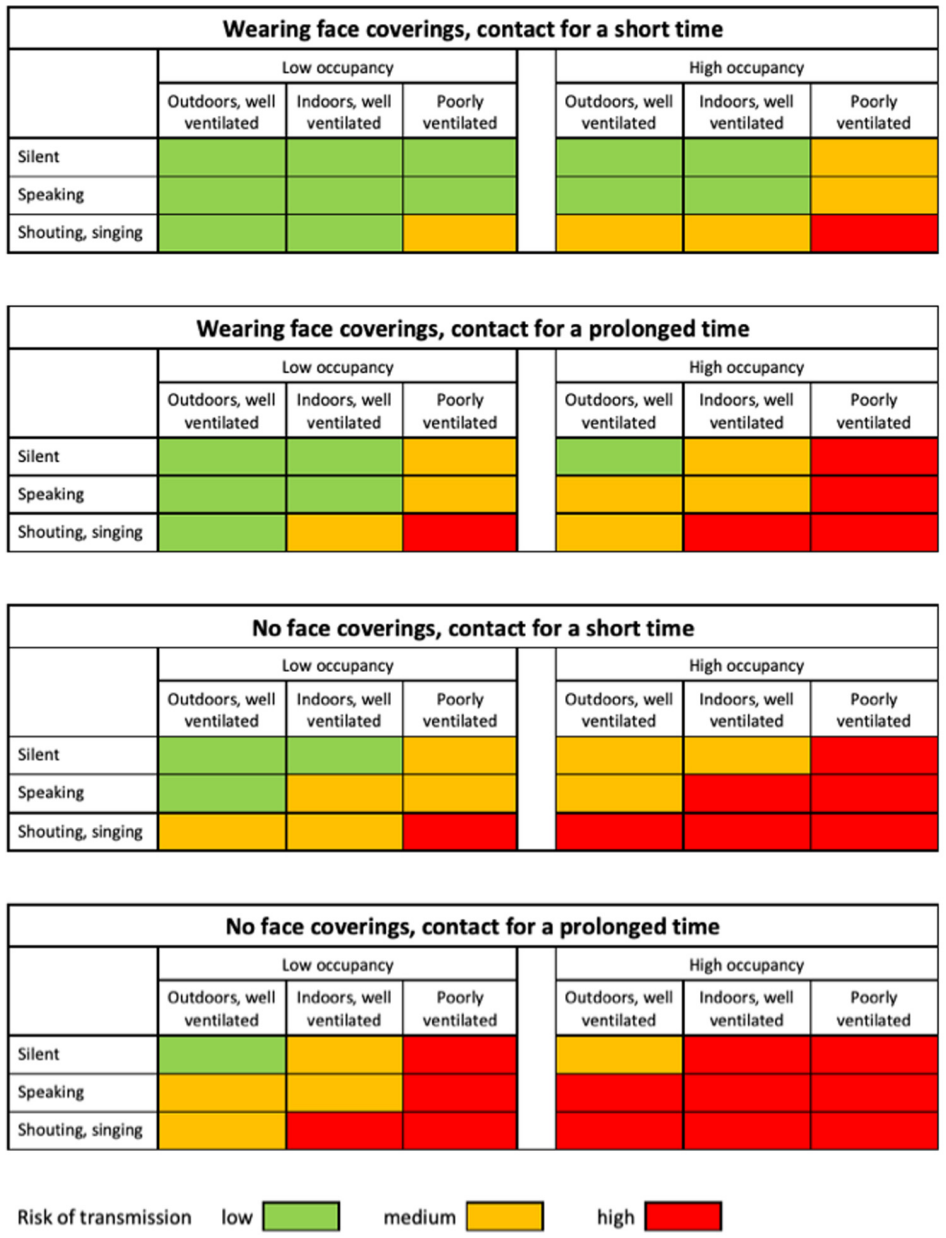
Risk of severe acute respiratory syndrome coronavirus 2 (SARS-CoV-2) transmission in different settings, assuming people are asymptomatic (adapted from 33 under Creative Commons licence).

Figure 2 summarises this information in a semi-quantitative way ^33^; a paper offering a quantified version of this diagram is available as a preprint ^34^.

In reality, however, university staff are often given a rigid separation distance to impose. It is important not to be overly reassured by such measures but instead take account of the multiple influences on transmission risk *.* Separating desks is a good idea, but also—and more importantly—those responsible for local policy should encourage people to get fully vaccinated, keep masks on, and speak quietly rather than loudly (and perhaps keep talking to a minimum while indoors). 

The fewer people who are physically present in the room, the lower the risk of transmitting the virus. This is partly because desks can be more spread out, but it is also because fewer breathing humans will be exhaling virus into the air. Ideally, a blended learning option should be offered in which those who wish to join the class remotely are supported to do so (especially if they or a household member are clinically vulnerable). Staggering the start dates of students does not appear to reduce on-campus transmission ^35^.

There is no evidence that introducing freestanding plastic screens (or other barriers between desks) reduces the risk of transmission or alters the benefit that is conferred by spacing desks, and such barriers may interfere with the effective circulation of clean air ^12^. Hence, universities should not attempt to install screens as a substitute for distancing or engineering controls.

### Deliver clean air by ventilation, filtration or ultraviolet (UV) inactivation

Heating, ventilation and air conditioning (HVAC) technologies and standards are designed to deliver clean air and thermal comfort to indoor spaces. The literature on HVAC uses the key concept of *air changes per hour* (ACH), and generally recommends 4 to 6 per hour equivalent for an average teaching room, achieved through natural or mechanical ventilation, air filtration or sterilisation (note that higher ACH rates are needed for some activities such as singing or gym ^36^). Another key metric for air quality when portable filtration units are used is the *clean air delivery rate* (CADR); these and other standards are explained in a recent review paper ^37^.

Ventilation in this context is defined as the intentional delivery of the outside air to a building’s indoor space. The obvious way to do this is to open windows (preferably on opposite sides of a room, or with a door open, to get a through draught). The effectiveness of opening windows depends on the design of the window and also on the weather. In one modelling study (currently a preprint ^38^), the most effective single intervention for reducing aerosols was natural ventilation through the full opening of six windows all day during the winter—a measure which led to a 14-fold decrease in cumulative dose of aerosol. This was more effective than universal use of surgical masks (which led to an 8-fold decrease). In the spring and summer, natural ventilation with windows fully open all day was less effective (2-fold decrease in cumulative dose). In the winter, partly opening two windows all day or fully opening six windows at the end of each class produced an approximately 2-fold decrease in cumulative dose of aerosols. In that study, opening windows during breaks only had minimal effect (≤ 1.2-fold decrease). The conclusion from this study is that if it is not possible to open windows more than a crack, a different way of cleaning the air is likely needed.

Whereas mechanical ventilation in domestic settings tends to occur through extractor fans (such as those in kitchens and bathrooms) or ceiling fans (often used as an alternative to air conditioning in hot climates), most mechanical ventilation in universities and colleges is through large systems which take in air through air handling units and supply and extract through a system of ducts and diffuser grilles. Lecture theatres and laboratories are generally mechanically ventilated via centralised HVAC systems. Hence it should not be assumed that if a space has no opening windows, it must be inadequately ventilated.

If indoor spaces are fitted with air conditioning systems, it is important to ensure that air which is removed is not recycled unfiltered (or inadequately filtered) back into that space ^12^. Air conditioning is not mechanical ventilation, though it may be linked to mechanical ventilation via large, central HVAC systems which both filter and heat (or cool) the air as needed. More problematic are isolated rooms fitted with their own local air conditioning systems, which are less likely to include any filtration and may give a false sense that the room is being ventilated.

The level of ventilation and occupancy in ventilated spaces can be approximated by measuring carbon dioxide (CO _2_) levels, since this is present in higher concentrations in exhaled air than in outdoor air. The higher the CO _2_ level in a room, the more exhaled air (and hence, potentially, the more virus) there is. Before the pandemic, indoor air quality standards were generally set around the goal of avoiding “sick building syndrome” (with symptoms such as headaches a sense of stuffiness, due to accumulation of multiple contaminants in the air) and clearing body odours and other smells. 

**Figure 3.**
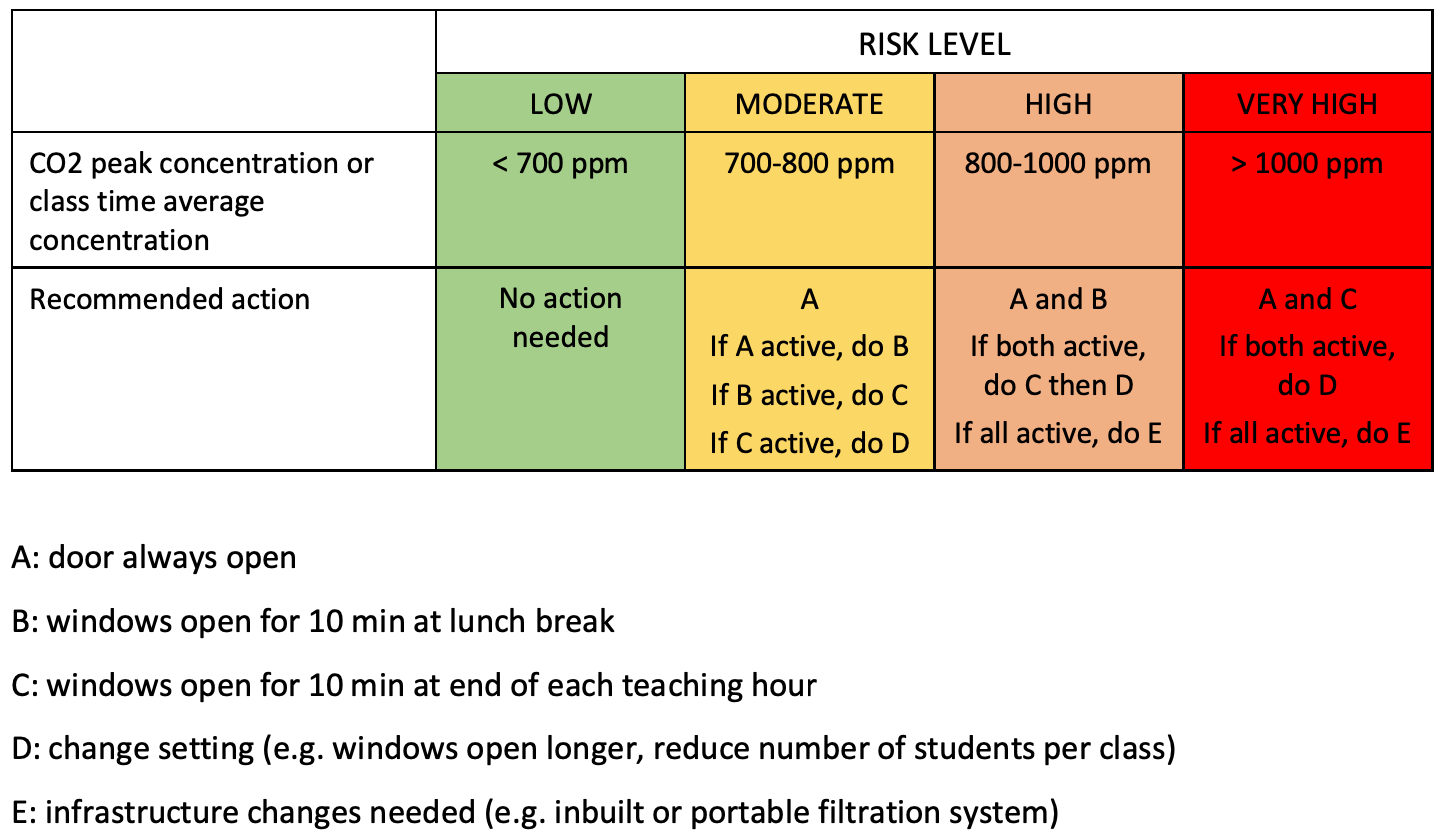
Risk classification scheme for carbon dioxide (CO _2_) levels in indoor air (adapted under Creative Commons licence from 40).

Whilst CO _2_ levels can be used to approximate the risk of COVID-19 transmission ^39^, they are only a proxy for this risk. With that caveat, some authors have suggested that CO _2_ levels might be used strategically in negotiations with employers ^40^. Figure 3 shows some suggested cut-off levels for denoting “low risk” (below 700 ppm), “medium risk” (700–800 ppm), “high risk” (800–1000 ppm) and “very high risk” (>1000 ppm), though other publications recommend slightly different cut-offs for these categories. Measures to address moderate risk include opening classroom doors and windows, opening windows between classes, and reducing the number of students in the classroom. If levels indicate “high risk” despite these measures, infrastructure changes (such as mechanical or portable air filters) are needed. 

Note that the cut-off values for unacceptable CO _2_ levels in Figure 3 are substantially lower than those in many official documents (e.g. from UK Health and Safety Executive, who recommend 1500 ppm ^41^). This is because the higher cut-off values were set historically for an entirely different purpose

When it is not possible or desirable to use ventilation (e.g. for energy efficiency reasons) to maintain clean air, other kinds of control are needed. There are two main kinds: an inbuilt mechanical filter (for which standards are expressed as the minimum efficiency reporting value (MERV) in the USA ^37^ or ISO 16890 in Europe) or a portable air cleaner fitted with a HEPA (high-efficiency particulate air) filter. Such filtration systems have been designed to remove particles of many different kinds and sizes (e.g. dust, pollen, smoke, bacteria, viruses).

The SARS-CoV-2 virus is approximately 100 nanometres (0.1 *μ*m) in diameter (though it is unlikely to travel naked so the size of particle to be filtered will be larger than this). The system installed should be efficient in the 0.1 to 1 *μ*m range. Of note to those in charge of supplying clean air to old-fashioned university or school buildings is this warning: *“most central mechanical systems were not designed for HEPA filters. Instead, these systems use filters on a different rating scale, minimum efficiency reporting value, or MERV, and typically use a low-grade filter (eg, MERV 8) that captures only approximately 15% of 0.3- to 1-μm particles, 50% of 1- to 3-μm particles, and 74% of 3- to 10μm particles. For infection control, buildings should upgrade to MERV 13 filters when possible, which could capture approximately 66%, 92%, and 98%, of these sized particles, respectively”* ^37^.

Upgrading from MERV8 to MERV13 filters (or the ISO equivalent) is potentially a rapid, affordable and effective intervention for universities and colleges in some settings, but higher-grade filters may induce a bigger pressure drop so unless the fan speeds can be increased, the ventilation rate may be inadequate.

Portable air filtration units fitted with HEPA filters are highly effective at removing aerosols in the 0.1 to 1 *μ*m range ^42– 45^. In the Villiers study described above, one HEPA filter was as effective as two windows partly open all day during the winter (2.5-fold decrease in cumulative dose of aerosols) while two HEPA filters were more effective (4-fold decrease) ^38^. A combination of interventions (masks along with natural ventilation *and* HEPA filtration) were the most effective, producing a 30-fold decrease in cumulative aerosol dose ^38^. Aerosol scientists have begun to develop and test home-made, low-cost box fans fitted with HEPA filters as a quick and effective solution for improving mechanical ventilation in poorly-ventilated spaces ^46^.

Ultraviolet (UV) light (from sunlight or radiation lamps) has been shown to destroy SARS-CoV-2 in numerous studies ^47^, though if this method is used it is important to select appropriate units that do not generate ozone. UV sterilization holds potential for enhancing safety in indoor spaces where risk of transmission is particularly high (e.g. hospitals, gyms). In a small before-and-after study published as a preprint, a combination of HEPA filtration *and* UV sterilisation was highly effective at removing bioaerosols (including but not limited to SARS-CoV-2) in a COVID-19 surge ward and intensive care unit in one hospital, though the contribution of the UV component to the result is unknown ^43^.

Electronic air cleaning systems, for example those which use ozone, are of no proven efficacy in reducing COVID-19 transmission ^48^; they currently have no place in preventing transmission of SARS-CoV-2. Air filtration does not remove CO _2_, so CO _2_ monitors cannot be used to monitor the quality of filtered air.

In the longer term, universities should consider the need for a *paradigm shift* in the design and ventilation of buildings, to improve air quality standards and ensure that all indoor spaces meet these through adequate ventilation, filtration or sterilisation ^36^.

### Test, trace and isolate while COVID-19 incidence is high

In the context of high incidence of COVID-19 and an unvaccinated or partially-vaccinated student population, frequent testing of asymptomatic staff and students along with contact tracing and support to isolate has been shown to reduce on-campus transmission substantially ^35^. While lateral flow device (LFD) tests can detect asymptomatic cases and break chains of transmission, this measure depends on the efficacy of efforts to track and trace contacts and maintain and support the isolation of infected individuals. Anyone who is symptomatic should isolate immediately and take a gold-standard polymerase chain reaction (PCR) test, irrespective of the status of their LFD test. Universities should ensure clear and consistent communication on this matter as confusion still abounds.

Anyone with symptoms, even if they are perceived to be “just a cold”, should isolate immediately, and a negative LFD should never override the more accurate PCR test (see below). Note that the most common symptoms of delta infection (in order: headache, runny nose, sneezing, sore throat, loss of smell, fatigue) are different from the standard triad of cough, fever and shortness of breath which are still widely used to prompt PCR testing ^49^. Hybrid teaching options greatly facilitate immediate isolation, and students and staff with symptoms that may be due to COVID-19 should be supported to engage remotely if they are well enough to do so. Track and trace efforts are constrained by the specifics of the system. Universities may have additional information that can be harnessed to provide a further layer of safety. In the UK for example, individuals sharing a confined space for extended periods of time, for example, may not be contacted by the official Track and Trace system but could be identified via attendance lists.

Some authors have questioned the validity and expense of mass asymptomatic testing in populations where incidence of COVID-19 is low, due to the very large number of tests required to detect small numbers of positive cases ^50^. A recent modelling study suggests that as vaccination rates rise and the incidence of COVID-19 falls, the cost-benefit balance of frequent testing becomes less favourable ^16^. However, at the time of writing the UK is a long way from a low-incidence state and we strongly recommend maintaining asymptomatic testing. Below, we explain some of the science behind the tests.

Lateral flow devices (LFDs), which detect the presence of virus antigen in the nose and throat using a swab sample tested in a flow device (like a pregnancy test) ^51^. Multiple types of LFD test are available, and they are designed to test people (perhaps repeatedly) who are not displaying overt COVID symptoms. LFD tests are all highly specific i.e. they are very unlikely to give a positive result if the person is not infected. But LFD tests are not particularly sensitive (i.e. less able to detect very small quantities of the virus) compared with the gold standard PCR (polymerase chain reaction) tests. This means that testing negative on an LFD is not a “green light” i.e. it does NOT guarantee that the individual is not infected with SARS-CoV-2, so they should continue to practice mitigations as advised. On the other hand, testing *positive* on an LFD means it is highly likely the person *is* infected (it is a “red light”, indicating that they are potentially infectious). Such individuals should self-isolate immediately, report the positive test, and order a confirmatory PCR test as soon as possible. A positive LFD test should trigger a call from the Track and Trace service.

Whilst LFD tests are used mainly in people without symptoms, they are actually more likely to be positive if the infected person is symptomatic (probably because such people have higher levels of the virus) ^52^. However, people with a positive LFD may well be infectious despite lack of symptoms—hence the value of these tests in identifying infectious cases (who should then isolate) and reducing the chance of a super-spreader event on campus. LFD tests also tend to reflect past infection (they are more likely to be positive 2 weeks after the onset of symptoms than on the day symptoms appear) ^52^.

In sum, the on-site LFD testing established at many UK university sites appears to be evidence-based (though not scientifically perfect) and its regular, frequent use is recommended while the incidence of COVID-19 remains high. Those with symptoms also need a PCR test.

### Clinically vulnerable staff and students

Universities and colleges have a duty of care to their staff and students. They must provide a safe environment for learning, teaching, and working. If a person has a condition or risk state which makes them vulnerable to COVID-19 and its complications, the institution must take account of this. Increased vulnerability to COVID-19 occurs in people who are immunosuppressed (including those on medication which suppresses the immune system, and pregnant women), those with certain long-term conditions, older age groups, some minority ethnic groups and those who are overweight. These risk groups were considered in detail in the Independent SAGE report ^1^.

The evidence supports a policy of vulnerable groups (whether staff or students) being supported to work from home if possible while the incidence of COVID-19 is high. If they must enter indoor spaces they should be advised to wear a respirator mask for self-protection, and it is particularly important for others in the room to wear a mask to maximise source control. If clinically vulnerable people are required to enter indoor spaces, those spaces should be adequately ventilated (confirmed using CO _2_ levels) or have high-quality air filtration systems (MERV13 or HEPA) installed.

### Interventions for which there is no evidence (“hygiene theatre”)

We found no scientific evidence to support taking temperatures, sanitising hands before entering the classroom (though washing hands when they are dirty and after going to the lavatory is of course a general hygiene measure), restricting the sharing or exchange of fomites (i.e. potentially contaminated objects such as pens, paper, books or other study materials), wearing face visors, or separating desks with plastic screens. Such “hygiene theatre”, which links to a discredited hypothesis that the virus is spread mainly or exclusively by droplets ^53^, could potentially distract staff and students from measures which do work.

In relation to sanitising, hand hygiene is recognised good practice for the prevention of many infectious diseases, so it should not be dismissed or discouraged (but equally, should not be over-emphasised). In relation to fomite transmission, a large Brazilian study detected no SARS-CoV-2 virus on over 400 samples of mask fronts, cell phones, paper money or card machines during a wave of the pandemic ^54^. In other words, there is some evidence *against* the importance of fomite transmission. However, since the mode of transmission remains contested, it would seem sensible to discourage widespread sharing of pencils, books and other objects among students.

## Conclusion

The key to effective prevention of COVID-19 is acknowledgement of its predominantly airborne mode of transmission. Many widely-promoted measures—hand sanitising, strict 1- or 2-metre distancing, fomite precautions—wrongly assume an exclusively droplet mode of transmission assume and are therefore ineffective. Such thinking also dominates the thinking of senior management and many staff and students.

Acknowledging the importance of airborne transmission should lead to policies such as: a) masking at all times while indoors, with encouragement to wear higher-grade respirators for best protection (especially if clinically vulnerable); b) continuing attention to physical distancing but in a way that does not assume that a particular interval between desks makes the space “safe”, and using additional measures (joining remotely, cohorting, frequent breaks) to reduce crowding and time spent indoors; c) a greater focus on engineering controls (ventilation and/or filtration of air). In addition, university and college staff should encourage and facilitate vaccination, attend to testing and tracing, and be ready to instigate tighter controls (e.g. return to online teaching) if case numbers rise.

These measures should be implemented and evaluated. Monitoring of metrics such as CO _2_ levels and room occupancy rates may provide staff with hard data with which to negotiate with management.

## Data availability

No data are associated with this article.
