# Supplementary material for: Rapid evidence review to inform safe return to campus in the context of coronavirus disease 2019 (COVID-19)
Source: Wellcome Open Res. 2021 Oct 20;6:282. [Version 1] doi: 10.12688/wellcomeopenres.17270.1 (PMC8567688; doi:10.12688/wellcomeopenres.17270.1)
Supplement: Peer review history

# Peer review: Reviewer response for version 1

**DOI:** 10.21956/wellcomeopenres.19088.r46570

**Published:** 2021-11-03

**Authors:** Michelle Ananda-Rajah

**Affiliations:** 1 Department of Infectious Diseases, Alfred Health and Central Clinical School, Monash University, Melbourne, Vic, Australia

Excellent and timely review with no major concerns. The following are suggestions that may enhance the manuscript:

- Worth including a statement on mandating vaccination which some universities in the US (Yale I think) and Australia have done. This intervention has greatly increased uptake of vaccination in younger people and staff.
- Please further clarify page 4 para 2 "new cases increased by only 1.9 fold... 60%". It is difficult to understand, please revise. 
- To remain silent while unmasked is not pragmatic nor feasible in a University setting. The credibility of the paper could be called into question if the measures proposed are not implementable. Consider making this a suggestion or placing a higher emphasis on HVAC improvements.
- Please elaborate as to why natural ventilation with the windows fully open in spring and summer was less effective accounting for a 2 fold decrease only. What is the likely basis for this result?
- A CO _2_ ppm upper limit of 1500 ppm was set for another purpose, please mention this other purpose because it may be a barrier otherwise to the adoption of a lower threshold.
- Re LFD testing in asymptomatic people: how often should this be done and would it vary depending on community transmission rates.
- Consider a box summarising key interventions. 

Is the review written in accessible language?

Yes

Are all factual statements correct and adequately supported by citations?

Yes

Are the conclusions drawn appropriate in the context of the current research literature?

Yes

Is the topic of the review discussed comprehensively in the context of the current literature?

Yes

Reviewer Expertise:

health services research, artificial intelligence, clinical leadership

I confirm that I have read this submission and believe that I have an appropriate level of expertise to confirm that it is of an acceptable scientific standard.

# Peer review: Reviewer response for version 1

**DOI:** 10.21956/wellcomeopenres.19088.r46604

**Published:** 2021-11-01

**Authors:** Stephen Duckett

**Affiliations:** 1 Health Program, Grattan Institute, Carlton, Vic, Australia; 2 The University of Melbourne, Melbourne, Vic, Australia

This is an excellent and comprehensive review of the evidence relevant to improve the safety of return of students to universities and schools in the context of the continuing transmission of SARS-CoV-2. The review should be augmented to consider more explicitly a number of issues:

**Vaccination:**

- The review is written specifically for a United Kingdom audience, and it is perhaps for that reason that it does not mention universities or schools requiring vaccination of students or staff. In Australia, for example, in some states, government regulations effectively require that only vaccinated people are able to return to  campus. A number of universities have also introduced mandatory vaccination of the staff and students who are to be on campus. This obviously requires consultation with industrial organisations, but some reference to this issue would be useful to ensure a comprehensive review of options.

**Physical distancing:**

- The review appropriately refers to the droplet induced theories about physical distancing. This section should be broadened to include stronger consideration of issues relating to remote and hybrid working, cohorting, and workplace bubbles. There may be limited published evidence on these strategies, and for this reason they may have been excluded, but these are strategies that universities and schools are considering and have a logical basis.
- Separation of cohorts is an effective way of reducing the likelihood of spread from one group to another. For example, if a university or school has multiple offerings of the same subject, it may introduce rules to require students not to swap between offerings in a term/semester. Similarly, it might encourage staff work on campus only on designated days to minimise overlap between staff cohorts.
- Risk of transmission is maximised when people are congregating in small areas such as waiting to use lifts/elevators. In administrate workplaces. it may be useful to consider encouraging staggered start times to minimise congregation in lift/elevator waiting areas. Similarly, consideration should be given to dispersal strategies for students leaving large lecture theatres, including encouraging use of stairs. The emphasis on masks is appropriate but could be augmented by other strategies.

**Testing:**

- The paper discusses the importance of rapid testing but does not discuss how to encourage people to respond if they test positive. This includes for staff, ensuring that they are paid while isolating. Special provisions may be required for casual and temporary contract staff.
- Some staff will be able to continue to work remotely, and that should be encouraged, and arrangements should already be in place for that to occur.
- The review does not discuss the frequency of testing — whether people should be tested every day before attending school/university, every second day, etc. There may be evidence on optimal testing frequency that should be included. On a related matter, there is no mention of managing the risk of staff who are employed in multiple organisations because of casualisation of the workplace. Multiple employment increases the risk of transmission from one workplace to another and universities and schools may wish to attempt to limit the number of separate workplaces that casual staff physically attend.

Is the review written in accessible language?

Yes

Are all factual statements correct and adequately supported by citations?

Yes

Are the conclusions drawn appropriate in the context of the current research literature?

Yes

Is the topic of the review discussed comprehensively in the context of the current literature?

Yes

Reviewer Expertise:

Health policy, including policies about SARS-CoV-2

I confirm that I have read this submission and believe that I have an appropriate level of expertise to confirm that it is of an acceptable scientific standard.

# Peer review: Reviewer response for version 1

**DOI:** 10.21956/wellcomeopenres.19088.r46568

**Published:** 2021-10-25

**Authors:** Stefan J. Marciniak

**Affiliations:** 1 Cambridge Institute for Medical Research, University of Cambridge, Cambridge, UK

This review aims to provide a timely synthesis of the literature relevant to SARS-CoV-2 transmission in an educational setting and of the available mitigation strategies that might be employed to facilitate a return of in-person university teaching.

 This is an ambitious piece and will be of value to those involved in delivering higher education during the pandemic. Helpfully, the authors critically evaluate a wide variety of potential mitigation strategies, highlighting those supported by published evidence, while importantly identifying those for which the evidence does not support continuation. In the former “effective” category, the authors include vaccination, mask wearing, physical distancing, engineering solutions (room ventilation and air filtration), test/trace/isolation, and blended learning. Approaches that the authors conclude to be “ineffective” include strict hand hygiene, restricting spread via fomites, the wearing of face shields, and the separation of individuals by plastic screens.

 The article is clear, well-written, and makes useful evidence-based recommendations. The authors may wish to consider the following critical comments, which might help to improve the article further. In particular, in their effort to provide a useful synthesis of the evidence, at times the distinction between published literature and their interpretation becomes unclear. It might be helpful, and would strengthen the review, if this distinction were to be made more obvious.

**Methods section:**

- The methods section describes a search of PubMed in September 2021 for the terms “SARS-CoV-2”, “COVID-19”, “transmission”, “mitigation”, “school[s]” and “university/ies”, restricted initially to reviews. These were examined for relevant papers by “snowball searching”. It would be helpful to know the total number of articles assessed and to be provided with more detail about the processes and metrics used to evaluate their quality. Would a PRISMA diagram be valuable in this regard? The authors refer to the screening of articles, in part, by considering the impact factor of the journals in which they were published. While impact factors have some value as a heuristic for quality, this approach also has limitations. It would be reassuring to learn how the threshold for impact factor was set and whether this was corrected for journal type, for example, clinical vs non-clinical journals. It would also be valuable to provide more detail on the methodologies employed to avoid the introduction of any bias. This might be provided in the Methods section or in a dedicated “limitations” section.

**Main findings section:**

*General comments:*

- This section provides a narrative synthesis of the above search. It is clearly written and generally well-referenced. In places, however, the referencing is less extensive and it is not always clear if this reflects a deficiency in the published literature. More clarity on this would be helpful in allowing readers to evaluate the validity of the recommendations. It might also be helpful to provide a quantification of the strength of the recommendations, perhaps along the lines of the GRADE (Grades of Recommendation Assessment, Development and Evaluation) approach used in clinical guidelines. ^1^

*Specific comments:*

- It is stated that “.. *this 90% cut-off is based on a single preprint modelling study [16] that has yet to be peer-reviewed*.” Would the authors double check that this statement is true? The article appears to have been published on 31 August 2021 in *Annals of Internal Medicine*, although at the time of writing (24/10/2021), its status has not been updated on medRxiv.
- It is stated, “ *One key measure for improving safety is to make it very easy for people to get a vaccination on campus—for example by locating vaccination hubs close to settings frequented by target groups (e.g. outside lecture theatres or dining halls) and not requiring paperwork that people are unlikely to be carrying*.” This is an important point, but is this an opinion of the authors or are there empirical studies to support it? Is it possible to make clearer the distinction between available data, knowledge gaps, and opinion?
- It is stated, “ *There have been claims that randomised controlled trial (RCT) evidence is the only “robust” way to test the impact of masks. This is incorrect, because most such RCTs are designed only to test the hypothesis that the mask protects the wearer over a short period.*” Again, this is very important. Is it possible to provide citations for the relevant articles? Similarly, elsewhere in this section, comments are made such as “ *Perhaps the most persuasive argument for masks in the university context is that if everyone wears one, there is a much lower risk that teaching will need to return to online as a result of rising case numbers*”. These comments are important and would be strengthened if published evidence could be cited. However, if this is interpretation, would it be valuable to separate the literature review from the interpretation?
- It is stated, “ *The benefit of mask wearing by all is not dependent on the size of a group, so suggestions that masking is needed only above a certain occupancy threshold means that unmasked smaller groups would carry a preventable risk (and also provide a false sense of security)*.” Are there published studies available to support this?
- Figure 2 encodes “risk of transmission” graphically by using a red/amber/green colour scheme. Would it be possible to amend the figure to be more accessible for those with deficiencies in colour vision? I suspect that in its current form it would be challenging for readers with deuteranopia or protanopia.
- In the section on testing, the authors might wish to consider a discussion of mass asymptomatic PCR testing of undergraduates, which has been shown both to be possible and effective in the university setting, for example, through the testing of pooled screening-samples from groups of 10 to 12 students followed by individual testing of positive groups. This has recently been reviewed. ^2^
- I do not feel able to comment usefully on the engineering solutions section, which falls outside my areas of knowledge.

Is the review written in accessible language?

Yes

Are all factual statements correct and adequately supported by citations?

Partly

Are the conclusions drawn appropriate in the context of the current research literature?

Yes

Is the topic of the review discussed comprehensively in the context of the current literature?

Yes

Reviewer Expertise:

Respiratory medicine, including COVID-19; cell biology, ER stress

I confirm that I have read this submission and believe that I have an appropriate level of expertise to confirm that it is of an acceptable scientific standard, however I have significant reservations, as outlined above.

## References

[1] : Grading quality of evidence and strength of recommendations. *BMJ* .2004;328(7454) : 10.1136/bmj.328.7454.1490 1490 10.1136/bmj.328.7454.1490 15205295PMC428525

[2] : Assessing COVID-19 Prevention Strategies to Permit the Safe Opening of Residential Colleges in Fall 2021. *Ann Intern Med* .2021; 10.7326/M21-2965 10.7326/M21-2965 34461034PMC8422997
